# Effect of Timing on Visual Outcomes in Fovea-Involving Retinal Detachments Verified by SD-OCT

**DOI:** 10.1155/2020/2307935

**Published:** 2020-02-21

**Authors:** Reinhard Angermann, Nikolaos E. Bechrakis, Teresa Rauchegger, Marina Casazza, Yvonne Nowosielski, Claus Zehetner

**Affiliations:** ^1^Department of Ophthalmology, Medical University Innsbruck, Innsbruck, Austria; ^2^Department of Ophthalmology, Paracelsus Medical University Salzburg, Salzburg, Austria; ^3^Department of Ophthalmology, University Hospital Essen, Essen, Germany

## Abstract

**Purpose:**

To investigate the impact of surgical delay after the objectivation of the foveal status by spectral-domain optical coherence tomography (SD-OCT) on visual outcomes in patients with rhegmatogenous retinal detachment (RRD) with foveal involvement.

**Methods:**

A retrospective dataset analysis of 508 eyes of 504 consecutive patients with primary RRD was performed. The primary outcome measure was the best-corrected visual acuity as a function of time between the assessment of the foveal status with SD-OCT upon initial examination at the department and RRD repair.

**Results:**

In total, 188 eyes (37.0%) had a complete foveal detachment and 31 (6.1%) eyes had a bisected fovea by the retinal detachment. A hundred eyes with total foveal detachment received surgery within 24 h and 65 eyes between 24 h and 72 h. Visual outcomes for eyes with detached fovea were significantly better when treated within 24 h (0.47 ± 0.39) compared with those treated between 24 h and 72 h (0.84 ± 0.66; *p*=0.01) after objectivation of the foveal status with SD-OCT. Pars plana vitrectomy was performed in 174 (92.6%) eyes and scleral buckling surgery in 14 (7.4%) eyes with complete foveal involvement of RRD.

**Conclusions:**

Our findings suggest improved visual outcomes for patients receiving surgery within 24 h after a definitive diagnosis of fovea-involving RRD compared to surgical interventions that were further delayed.

## 1. Introduction

In the management of foveal detachment, the time until surgery is the most important prognostic factor for final visual acuity (FVA) after reattachment [1, 2]. Several factors, such as height of foveal detachment, age, and preoperative visual acuity, have been determined to have an impact on visual outcomes. However, after diagnosis, lag time until surgery remains the only variable that can be influenced.

The duration of tolerable delay time for surgical intervention is a controversial and unresolved issue in the literature. The recommendation of maximum lag time until surgery varies from 1 to 10 days [1–5]. One common finding among previous studies investigating the delay of surgery is the extensive variation of FVA after repair of macula-off rhegmatogenous retinal detachment (RRD). However, preoperative characteristics can only partly explain the great differences in FVA.

Accurate dating of foveal detachment is a limitation faced by all prior studies addressing surgical timing of macula-off RRD. The definition of macula-off duration by subjective symptoms such as central vision loss lacks objectivity. In fact, a study by Ricker et al. [6] found that one-third of patients with assumed foveal attachment, as determined by clinical symptoms and fundoscopy, showed foveal detachment by optical coherence tomography (OCT). This finding indicates a great variation in subjective symptoms between individuals.

The main purpose of this study was to determine the effect of surgical timing on visual recovery of patients receiving surgery within 3 days after a definitive diagnosis of fovea-involving RRD by spectral-domain optical coherence tomography (SD-OCT).

## 2. Materials and Methods

### 2.1. Dataset and Patients

Data were documented utilising a structured electronic database for patients with primary RRD. Implementation and application of the RRD dataset was validated by the Department for Strategic Quality Management of the University Clinic Innsbruck, Innsbruck, Austria. The collected data included information about sex, eye, lens status, refraction, best-corrected visual acuity (BCVA), presence of pathological myopia, history of traumata and complicated cataract, previous surgery, and laser therapy as well as the presence of vitreous bleeding, characteristics and location of retinal tears, extent of retinal detachment and affected quadrants, foveal detachment, lag time from SD-OCT at the department until surgery, proliferative vitreoretinopathy (PVR)-classification, and surgical methods. Patients with vitreous haemorrhage were excluded from the statistical analyses of BCVA to prevent the potential bias. During hospitalisation, ophthalmological examinations were performed by retinal specialists. Spectral-domain optical coherence tomography (OCT Spectralis® Heidelberg, Germany) was performed in all cases to verify whether the fovea was attached.

A total of 508 eyes of 504 consecutive patients diagnosed with primary RRD, including severe PVR grades B and C, and treated at the Department of Ophthalmology, Medical University Innsbruck, Innsbruck, Austria, were included in the study. Patients directly presented to the emergency department of the clinic or were referred from private ophthalmologists or other hospitals. The exclusion criteria included previous vitreoretinal surgery, posterior uveitis, or a history of penetrating trauma. Data were collected in the electronic medical record system of the department and were audited retrospectively via implementation into a standardised RRD database. All data were anonymised prior to analysis.

This retrospective study was approved by the Local Committee for Medical Research Ethics, and consent was waived by the Institutional Review Board of the Medical University Innsbruck, Innsbruck, Austria. All procedures performed involving human participants were in accordance with the ethical standards of the institutional and/or national research committee and with the 1964 Helsinki Declaration and its later amendments or comparable ethical standards. The Innsbruck Rhegmatogenous Retinal Detachment Register and the electronic dataset were accredited by the administrative department for quality and risk management of the Landeskrankenhaus–University Clinic Innsbruck, Innsbruck, Austria.

### 2.2. Clinical Assessment and Grading

Electronic medical records were used for the outcome analysis. Vitreoretinal surgery was conducted at a tertiary medical centre based in the federal state of Tyrol.

Surgeries were performed under retrobulbar or general anaesthesia. Surgical methods included PPV, SB, or combined PPV-SB. Patients who underwent cataract surgery combined with PPV or PPV-SB were also included. PPV involved standard 20-gauge (G) and transconjunctival 23-G, 25-G, and 27-G trocar-guided instrumentation, use of a noncontact wide-angle viewing system (BIOM, Oculus GmbH, Wetzlar, Germany), endolaser or transscleral cryocoagulation, fluid-air exchange with air or gas (SF6, C3F8, or C2F6), or silicone oil (5000 centistokes) as endotamponade. Heavy liquids (perfluorocarbon) and retinotomies for internal drainage were optional elements of the surgical procedures. PPV-SB consisted of PPV combined with an encircling 2.5-mm or 4-mm band (FCI, Paris, France). SB surgery included a localised silicone (5 mm or 7.5 mm) sponge, encircling 2.5-mm band, or an encircling band combined with a localised silicone (5 mm or 7.5 mm) sponge. During buckling procedures, cryopexy was used with or without external drainage.

### 2.3. Statistical Analysis

Demographic data and baseline findings are presented as the number of patients with percentages, while continuously distributed data are reported with their mean and standard deviation (SD). The Kolmogorov-Smirnov test was employed to test all variables for normal distribution. Unpaired sample *t*-tests and analysis of variance for normally distributed data were employed. Nonnormally distributed data were compared with MannWhitney *U* test. Categorical data were compared using a chi-squared test and Fisher's exact test. The Pearson's correlation or the Spearman's rank correlation coefficient was calculated to analyse correlations between parameters. All *p* values <0.05 were considered significant. Statistical analyses were performed using SPSS Statistics® (IBM, Armonk, NY, USA).

## 3. Results

Data of 508 consecutive eyes from 504 patients were included in the study. The mean age was 60.2 (±13.8) years, and 322 (63.4%) of the patients were men. At initial examination, a total of 219 (43.1%) eyes presented with fovea-involving detachment, 188 eyes (37.0%) had a complete foveal detachment, and 31 (6.1%) eyes had a fovea bisected by a retinal detachment. One-hundred and fifty-three (30.1%) eyes were observed to have more than one retinal tear. Further baseline characteristics are shown in detail in Table 1.

### 3.1. Visual Outcome of Patients with Fovea-Involving RRD

**Figure 1:**
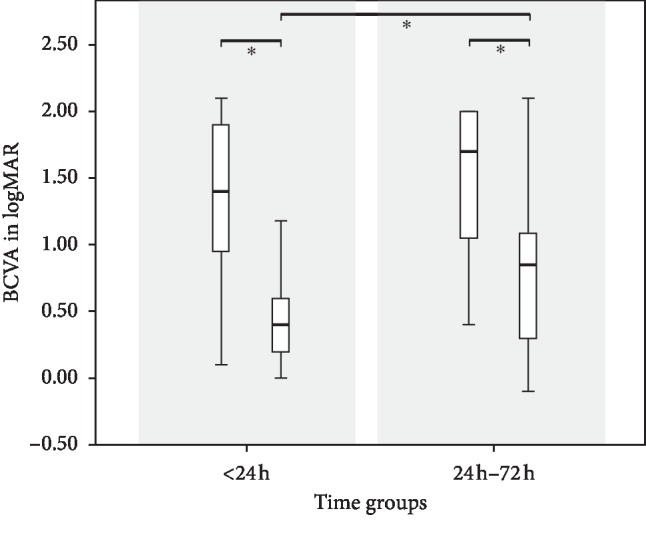
The best-corrected visual acuity (BCVA) of the fovea-involving primary rhegmatogenous retinal detachment at baseline and at follow-up examination, after surgical reattachment categorized into time groups. There was a significant increase in BCVA after surgery in both groups and a significantly better outcome in patients receiving surgery within 24 h compared to those receiving surgery 24 h to 72 h after diagnosis.

In Table 2, data are divided into three groups depending on the status of the fovea and further subdivided according to the time elapsed until the beginning of the surgery up to 72 h after SD-OCT assisted diagnosis. A comparison of mean BCVA before surgery and at follow-up was performed. At baseline of fovea-off RRD, there was no significant difference between the time groups. Among patients with a detached fovea, BCVA at follow-up was significantly improved in patients who underwent surgery within 24 h (0.47 ± 0.39) after initial presentation when compared with patients who were treated between 24 h and 72 h (0.81 ± 0.63; *p*=0.009) (see Figure 1) or between 24 h and 48 h (0.84 ± 0.66; *p*=0.009). No significant difference in outcomes could be detected between patients receiving surgery 24 h to 48 h and 48 h to 72 h after diagnosis (*p*=0.825).

## 4. Discussion

Detachment of the fovea has been identified as a negative prognostic indicator for visual acuity outcomes after surgical treatment for RRD. A major impact on the visual outcome of primary RRD is whether the detachment reached the fovea [7, 8]. As soon as central detachment is present, improvement of central vision often remains compromised on account of permanent functional damage to the fovea. Several studies have concluded that the final functional outcome after surgical repair of fovea-off RRD is time dependent [1–3, 9].

However, the accurate timing of foveal detachment is a major limitation faced by all studies investigating surgical timing in fovea-off RRDs [1, 2, 5, 9, 10]. Most studies investigating the influence of surgical timing used patient-reported beginning of central vision loss as a surrogate marker. Additionally, patient-reported history about the onset of central vision loss was documented with a rather inaccurate time frame using days or 24 h time periods as calculated units of time. Central vision loss represents a rather subjective methodology and may vary greatly between individuals. Even after biomicroscopic examination of the foveal status, the exact definition of macular involving RRD remains inaccurate. This consideration is supported by a study of Ricker et al. who showed that, without preoperative OCT scanning, 25 of 53 patients (47%) were classified as macula-involving RRD patients, whereas this number increased to 38 of 53 patients (72%) with preoperative OCT scanning. The authors therefore stress the importance of preoperative OCT scanning in the allocation of RRD patients [6].

Considering that the onset of visual symptoms does not correlate with the clinical manifest diagnosis of foveal detachment, we employed a stricter methodical approach for assessing foveal involvement of retinal detachment with SD-OCT in all cases. In our study, analyses were conducted using clinical data from the ophthalmological diagnosis of fovea-involving retinal detachment based on SD-OCT, rather than subjective clinical onset. Our findings suggested that surgery within one day after diagnosis of retinal detachment by SD-OCT resulted in better FVA compared to surgical interventions that were further delayed.

Our study outcome suggesting a fast intervention regarding fovea-involving RRD is further supported by the results of animal model studies, which reported that photoreceptor apoptosis was highest after 24 h following RRD onset, but subsequently declined after 72 h, progressively [11, 12]. These models suggest that the visual outcome of fovea-involving RRD would be best if treated within a day. However, most previous clinical studies' results are clearly not consistent with the results of animal models. Most studies noted no difference in visual outcomes in eyes repaired between 7 and 10 days after onset of central vision loss [10–13]. By contrast, a review of nine studies by Van Bussel et al. [2] reported a significant worsening of FVA after a duration of central vision loss greater than 3 days. These authors also reported that a major limitation of their review was the lack of sufficient data of FVA in patients receiving surgical repair within first 3 days of foveal detachment.

A recently published study by Frings et al. [1] found that the best FVA was achieved after surgical interventions within 3 days after central vision loss. Notably, no statistically significant difference in FVA could be seen in patients who obtained surgical treatment within the first 3 days. In contrast, a more recent study by Greven et al. [5] proposed a difference of FVA in patients receiving surgical intervention after a duration of 1 day of macular detachment compared to those treated after 3 days of central vision loss. However, both study groups stated that the outcome for surgical timing within the 3 days should be interpreted with great caution, as they had a low sample size of only 35 eyes. Furthermore, in their studies, they used written documentation of the history of the patient's subjective visual symptoms as a surrogate marker for the duration of retinal detachment involving the fovea. They considered that patient-reported symptoms are a rather subjective methodology and may vary between individuals, which they acknowledged as a limitation of their study. In addition, the rather inaccurate definition of foveal status and the timing of the subjective vision loss at the conception of the study protocols may have contributed to the lack of conclusive study findings regarding the influence of surgical timing of RRD surgery.

There are some limitations to the present study. Due to the small number of cases we were not able to draw any conclusions regarding the impact of time on FVA in the primary RRDs with bisected fovea. In some cases, complicated RRD might be the reason for a prolonged waiting time until surgery. The duration until surgery was defined as the time elapsed between the SD-OCT assisted objectivation of the foveal involvement of the RRD and surgical treatment and might include patients with late clinical recognition of central vision loss. Patients' history of onset of central vision loss was not specific enough for proper statistical evaluation. However, in contrast to the varying subjective notification of central vision loss in written documentation of previous studies, our study protocol allowed us to note the exact lag time between OCT-assisted diagnosis and surgical intervention. This potential bias strengthens the proposed narrow time window after OCT-assisted diagnosis of foveal detachment and cements the importance of fast surgical intervention, especially in cases where the clinical onset of central vision loss is unsure. The present findings show that fast surgical treatment after diagnosis is paramount in order to achieve best visual outcome.

The strengths of this study include that all patients underwent diagnosis and treatment at one university hospital. OCT was performed to analyse fovea involvement in all cases. Our definition of lag time until surgery and data of visual recovery will help clinicians to decide to which extent fovea-involving RRD should be considered as a medical urgency. Future research should focus on further clarifying the FVA outcomes in cases of fovea-involving RRD patients who receive surgical treatment within 3 days.

In conclusion, our findings suggest better visual outcomes for patients receiving surgery within 24 h after the definitive diagnosis of fovea-involving RRD with SD-OCT compared to surgical interventions that were further delayed.

## Figures and Tables

**Table 1:** Descriptive baseline data.

	*n* (%) or mean (±SD)
Male/Female	322 (63.4)/186 (36.6)
Age	60.17 (±13.8)
Phakic/Pseudophakic	300 (59.1)/208 (40.9)
Size of RRD (n of clock hours)	4.77 (±2.5)
<3 h	164 (32.3)
>3–<6 h	251 (49.4)
>6 h	93 (18.3)
Macula status	
Attached	275 (54.1)
Bisected	31 (6.1)
Detached	188 (37.0)
NA	14 (2.8)
Retinal tear	1.55 (±1.52)
>1 tear	153 (30.1)
Giant tear	12 (2.4)
Oradialysis	6 (1.2)
Superior temporal	303 (59.6)
Superior nasal	292 (57.5)
Inferior nasal	232 (45.7)
Inferior temporal	232 (45.7)
Vitreous haemorrhage	60 (11.8)
Pathological myopia (>6 diopters)	82 (16.1)

h, hours; *n*, number of patients; NA, not available; RRD, rhegmatogenous retinal detachment; SD, standard deviation.

**Table 2:** Comparison of the best-corrected visual acuity between patients who underwent rhegmatogenous retinal detachment repair surgery with a lag time of less than 24 h and those with a lag time of 24–72 h categorized based on the foveal status.

	Baseline BCVA logMAR		*p*-value	Final BCVA logMAR		*p*-value
Foveal status	<24 h (SD; *n*)	24–72 h (SD; *n*)		<24 h (SD; *n*)	24–72 h (SD; *n*)	
Attached	0.46 (0.51; 181)	0.57 (0.62; 74)	0.14	0.22 (0.32; 87)	0.30 (0.44; 30)	0.24
Bisected	0.72 (0.50; 22)	0.75 (0.60; 6)	0.90	0.29 (0.31; 8)	0.52 (0.61; 4)	0.39
Detached	1.48 (0.55; 100)	1.50 (0.56; 65)	0.80	0.47 (0.39; 40)	0.81 (0.63; 24)	0.009

BCVA, best-corrected visual acuity; h, hours; logMAR, logarithm of minimum angle of resolution; *n*, number of patients; SD, standard deviation.

## Data Availability

The data used to support the findings of this study are available from the corresponding author upon request.
